# Assessing Acceptability: The Role of Understanding Participant, Neighborhood, and Community Contextual Factors in Designing a Community-Tailored Cooking Intervention

**DOI:** 10.3390/nu16030463

**Published:** 2024-02-05

**Authors:** Nicole Farmer, Ralph Tuason, Kimberly R. Middleton, Assumpta Ude, Gladys Tataw-Ayuketah, Sharon Flynn, Narjis Kazmi, Alyssa Baginski, Valerie Mitchell, Tiffany M. Powell-Wiley, Gwenyth R. Wallen

**Affiliations:** 1 Translational Biobehavioral and Health Disparities Branch, The National Institutes of Health, Clinical Center, Bethesda, MD 20892, USA; ralph.tuason@nih.gov (R.T.); middletonk@cc.nih.gov (K.R.M.); narjis.kazmi@nih.gov (N.K.); alyssa.baginski@som.umaryland.edu (A.B.); gwallen@cc.nih.gov (G.R.W.); 2 Clinical Center Nursing Department, The National Institutes of Health, Bethesda, MD 20892, USA; assumpta.ude@nih.gov (A.U.); gladys.tataw-ayuketah@nih.gov (G.T.-A.); sharon.flynn@nih.gov (S.F.); 3 Social Determinants of Obesity and Cardiovascular Risk Laboratory, Cardiovascular Branch, Division of Intramural Research, National Heart, Lung and Blood Institute, Bethesda, MD 20892, USA; valerie.mitchell@nih.gov (V.M.); tiffany.powell-wiley@nih.gov (T.M.P.-W.); 4 Intramural Research Program, National Institute on Minority Health and Health Disparities, Bethesda, MD 20892, USA

**Keywords:** cooking intervention, community tailored, African American, food environment, acceptability, mixed methods

## Abstract

Background: Cooking is an identified dietary strategy that is positively associated with optimal diet quality. Prior to initiating cooking interventions, evaluating the prospective acceptability of the intervention among community members living within low food access areas and understanding geospatial food shopping locations may aid in designing community-tailored interventions. Methods: A sequential mixed methods study was conducted to determine the prospective acceptability of a planned community-located cooking intervention among African American adults living in a low food access area and with at least one cardiovascular disease risk factor. A semi-structured guide was used to conduct five virtual focus groups. Qualitative data were analyzed using thematic analysis and validated through participant check-in interviews. Survey responses were analyzed based on descriptive data. Geospatial analysis of participant locations that were reported for food shopping was conducted to show food environment utilization. Results: Focus groups with study participants (n = 20, all female, mean age 60.3, SD 9.3, mean cooking frequency per week 4.0, food insecure n = 7) were conducted between March and April, 2021. Thematic analysis of the focus group transcripts identified five main themes as follows: (A) Barriers to Cooking (family and caregiving, transportation, COVID-19 pandemic, time availability, household composition); (B) Motivators for Cooking (family, caregiving, health, enjoyment, COVID-19 pandemic); (C) Strategies (food shopping, social support, social media, meal planning); (D) Neighborhood (gentrification, perceived safety, stigmatization, disparities in grocery stores); (E) and Acceptability of the Intervention (reasons to participate, barriers, recruitment, intervention delivery). Participant validation interviews confirmed the themes and subthemes as well as the illustrative quotes. Geospatial analysis showed a majority of locations were outside of the participants’ residential areas. Conclusions: Prospective acceptability of a community-tailored cooking intervention found that the planned intervention could be modified to address individual level factors, such as caregiving and health, community contextual factors, such as perceived safety, and the general health needs of the community.

## 1. Introduction

Dietary intake is a leading risk factor for death globally [1], yet a healthy diet in the U.S., defined as adherence to U.S. dietary guidelines, is one of the least prevalent health-promoting lifestyle factors among adults [2]. The low prevalence of a healthy diet may represent the inherent complexities involved in diet and food choice as well as the non-nutritional factors such as socio-economic status, environment, and psychological factors. In recent years, epidemiological data have identified cooking as a strategy positively associated with the promotion of dietary intake adhering to the U.S. dietary guidelines [3,4]. Proposed mechanisms to explain how cooking at home is associated with optimal dietary intake include the selection of foods supportive of a healthful diet, perception of diet quality, and less intake of meals that are not home-prepared [3,4]. Supportive of the epidemiological data that show a role for cooking in optimizing diet, cooking interventions are identified as successful strategies for dietary improvement and dietary behavior change among diverse populations [5,6,7,8,9]. Although dietary quality is poor among the majority of U.S. adults, there remains a disproportionate representation of diet-related chronic disease prevalence among adults self-identified as African Americans [10]. Cooking intervention studies including African Americans have reported improvement in self-reported dietary quality, but the overall inclusion of African Americans within clinical nutrition interventions remains a key gap area for addressing diet-related disease disparities [11]. Community-tailored cooking interventions may be required for the necessary inclusion of African Americans within intervention studies as African Americans represent racially categorized individuals who disproportionately live within racialized communities that serve as impediments to dietary quality as a result of adverse environmental and social factors [12,13,14,15].

One of the goals of cooking intervention research is to promote sustainable cooking behaviors at home. This goal is accomplished through the experiential learning of practical skills such as kitchen setup, meal planning and experiences, grocery shopping, nutrition label education, food storage, and food budgeting. The utilization and implementation of these skills can be influenced by factors that impact dietary intake, such as participants’ home composition, time availability, and occupation [16,17,18]. Moreover, cooking interventions in which food or meal kits are not provided require participants to conduct their own food provisioning potentially within low food access residential environments. Thus, an approach within cooking interventions to evaluate not only existing cooking behaviors (prior to the intervention) but also how participants utilize and access available food resources and retail environments may be necessary and further suggest a role for community-tailored or community-engaged interventions.

The optimization of interventions to promote the sustainability of behaviors, especially within adverse food environments, may be facilitated by formative research that utilizes evaluations of acceptability, participatory collaboration, and the identification of implementation barriers [7,8,9]. The acceptability of community research for potential participants can be essential for ethical considerations, participant recruitment, and participant retention. An operational definition of acceptability is the “perception among invited participants that the research design is favorable, agreeable or satisfactory” [19,20]. Acceptability can occur retrospectively (after an intervention) or prospectively (prior to an intervention) [21,22]. When conducted prospectively, acceptability may aid in tailoring an intervention to personal and social contexts. For instance, individual, household, or geographic circumstances may include if the intervention will address health concern(s), compatibility with lifestyle, and effectiveness in managing a problem for the participant [21,22]. Additionally, an understanding of prospective acceptability may be especially important for participants from low food access geographic areas or participants with chronic health conditions for which specific concerns regarding access and food availability would impact the implementation of the learned skills and the development of confidence in these skills [7,8,9].

This paper details the results of a sequential mixed methods study from the D.C. COOKS with Heart (community organizing for optimal culinary knowledge study with heart) study, a community-engaged intervention study among African American adults with cardiovascular disease risk factors who live within low food access areas within Washington, D.C. Using virtual focus groups, the study sought to obtain information about prospective acceptability and pre-intervention cooking behaviors as well as to conduct participatory collaboration to modify the intervention to best promote the implementation of learned dietary and cooking behaviors. Finally, acceptability results were validated using the community-participatory method of group member or participant check-in. 

## 2. Materials and Methods

### 2.1. Study Design 

D.C. COOKS with Heart is a dietary behavior intervention that builds on a long-term community-based participatory research (CBPR) study to design culturally specific, community-based interventions seeking to address cardiovascular risk factors within the Washington, D.C., metropolitan area. The CBPR study, which makes use of the Washington, D.C., Cardiovascular Health and Needs Assessment (NCT 01927783), is partnered with a community advisory board, which is the D.C. Cardiovascular Health and Obesity Collaborative (D.C. CHOC). Specific details of the Washington, D.C., Cardiovascular Health and Needs Assessment can be found in prior publications [23,24]. Consistent with CBPR principles, the overarching goal of the CBPR study is to conduct research that is consistent with the health needs and wants of the community. Prior assessment studies of the Washington, D.C., community found intervention research on physical activity and promoting a healthy diet to be identified as important by community members [23].

D.C. COOKS is a two-phase mixed methods study designed to assess the acceptability and feasibility of a cooking behavior intervention among African American adults who are at risk of CVD [25]. The primary behavior outcome of interest is that of home cooking behavior defined as the frequency of meals cooked at home and those with whom participants cook at home. A secondary dietary behavior change outcome is that of dietary intake. The agents of behavioral change are the study participants. The expected site for the intervention is a community-based kitchen site. A detailed description for both Phase 1 and Phase 2 of D.C. COOKS, including inclusion and exclusion criteria, is available in the published protocol paper [25,26]. Phase 1 of D.C. COOKS is designed to determine acceptability from community members who participated in moderated focus group sessions as well as a collection of surveys to assess demographic, cooking behavioral, health behavioral, and psychosocial factors. 

### 2.2. Study Population 

The study population of D.C. COOKS consists of African American adults (age ≥ 18) who reside within one of two neighborhoods (wards) in Washington, D.C., as follows: Ward 7 or Ward 8. From an economic and demographic standpoint, both Ward 7 and Ward 8 are comparable, with a median age of 35 years as well as a median household composition of 2.33 and 2.36, respectively. The median household income of African Americans living in both wards in 2023 was approximately $45,000 [27,28]. Within the Washington DC population, these communities have the highest prevalence of CVD-related risk factors, highest mortality rates for heart disease, and lowest life expectancy [29]. In addition, these communities are considered under-resourced in terms of accessibility to grocery stores/fresh produce food sources [30], which places residents in this community at risk of lower dietary quality. As a result of the low food access and the increased risk of chronic disease morbidity and mortality, Ward 7 and Ward 8 neighborhoods were selected for the D.C. COOKS study as the sites for intervention.

To conduct the acceptability phase of the study, recruitment of self-identified African American adults started in February, 2021. Recruitment occurred through an established community advisory board partnership in March and April of 2021, as described in detail the study of [31]. The research institution for D.C. COOKS is the National Institutes of Health Clinical Center. A single intramural institutional review board serves the NIH Clinical Center for oversight of research conduct and human subject protections. Due to COVID-19 social distancing restrictions, an amendment was submitted to the NIH Intramural IRB in December 2020 to conduct Phase 1’s study protocol focus groups virtually. The amendment was approved by the IRB. The recruitment of participants started in February 2021, and focus groups were conducted in March and April of 2021. The informed consent of participants was obtained virtually prior to the start of focus groups [31]. 

### 2.3. Data Collection and Procedures

Data procedures consisted of the collection of both quantitative and qualitative data. Electronic, self-administered surveys were used to measure demographic, residential food environment, cooking behavior, health behavior, and psychosocial variables. Consented participants were mailed a paper copy of all the survey questions to use during the focus group discussions as a reference for the proposed survey questions. Survey responses were collected through the use of the electronic tablets provided to each participant. The online survey was made accessible on these tablets by being “pinned” on the home screen. Additionally, a QR code was placed on the cover page of the paper copies of the surveys that were mailed to participants. The cover page also contained the participants’ unique study ID numbers to be used/entered for the purpose of the surveys. Participants were asked to complete all the survey questions prior to their scheduled virtual focus groups, and completion of surveys was verified by research team members prior to the start of each focus group. 

Five virtual focus groups were conducted with four participants in each focus group. The co-moderator of the virtual focus group also took on the role of scanning the virtual room for cues as to when other participants wanted to contribute or may have shown body language suggestive of having a response to a moderator question or to another participant’s comments.

All recruited participants (n = 20) were present at their scheduled virtual focus group. During the five focus groups, minor technical problems occurred including participants adjusting tablet volume or video screen for optimal viewing and the momentary loss of the shared screen when the moderator displayed shared content. All participants used the provided tablets and stands for the focus groups except for one individual who used their personal laptop.

A structured moderator guide was used for all the focus groups (Supplementary Materials). The guide included the topics of barriers and facilitators for cooking at home, motivators for cooking, the impact of the COVID-19 pandemic on food shopping, and feedback on the Phase 2 intervention. To elicit feedback, participants were provided an overview of the purpose and objectives of Phase 2, a proposed schedule of planned study visits and procedures, a sample educational video of a cooking skill from the intervention (how to dice a whole onion), two sample recipes, and a sample page from the proposed cooking diary. 

After each focus group, the research team conducted a debrief session regarding participant involvement and the topics discussed during the focus group. At the end of the last focus group, the debrief discussion included the research team’s observation of data saturation across the five groups in terms of the repeated topics being presented by the participants. The observation was based on research team members previously observing in-person focus groups in a previous study conducted within our CBPR studies, which involved participants from this same community [31]. These team members reported that the discussion and participant involvement were similar to those observed in previous in-person groups.

### 2.4. Thematic Analysis

Qualitative data collected were verbatim transcriptions and notes from research team members. Quality assurance of the verbatim transcripts was conducted by team members prior to analysis. Quality assurance included verifying the anonymization of transcripts and the use of audio recordings to verify inaudible statements on the transcript.

Thematic analysis was conducted using an iterative process among research team members (GW, SF, GT, AU, AB, NK). Research team members conducting the thematic analysis were predominately self-identified female within the range of ages from 25 to 60 years of age, and four team members self-identified from a minoritized ethnic group. None of the team members were prior or current residents of the study neighborhood. Thematic analysis was used to code the transcript data and to generate the resulting themes. Coding was initially done independently by research team members to highlight important phrases and sentences. Then, similar phrases and sentences were grouped, and codes were assigned. An iterative process ensued to discuss the results of the independent coding, and then a codebook was developed. Over consecutive meetings, the team met to refine the codebook, reconcile coding discrepancies, and develop themes. From the discussions, codes were created and finalized and then grouped into themes with subthemes. An independent research team member (KM) then evaluated all the themes, subthemes, and codes to validate them against the primary data source. During the process, when necessary, queries or disagreements were brought to the study PI (NF) in order to seek further consensus. All themes and codes generated were ultimately reconciled and agreed upon by all the research team members prior to continuing with focus group participant thematic validation. 

### 2.5. Validation Interviews by Participants

Following thematic analysis, validation interviews were conducted with focus group participants to optimize the rigor of the qualitative process by establishing the credibility, confirmability, dependability, and transferability of the thematic analysis results [32]. Prior to validation interviews, participants were sent a copy of the thematic analysis results including themes and representative quotes. All interviews were conducted over the phone and included the same members of the research study team (NF, RT, AB). During the interviews, research team members reviewed each theme and a set of representative quotes with the participants. Based on their recall from the focus group that they participated in, participants were asked to suggest changes to themes as they deemed necessary, to verify if the quotes selected were representative of the themes from their group, and to provide any suggested new themes or quotes. During the validation interviews, participants were additionally asked about the acceptability of the intervention and the contextual changes to the geographic location that may change perceptions of acceptability.

### 2.6. Construction of Participant-Identified Food Shopping Locations

**Figure 1:**
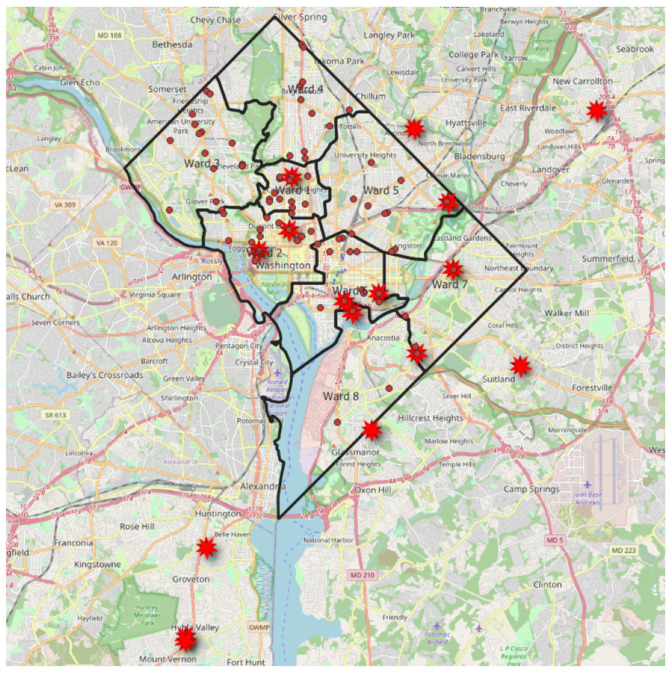
Food store locations (demarcated through red asterisks) as reported by Phase 1 DC COOKS focus group participants. Locations demarcated using red asterisks show food grocery store locations which participants travel to outside of their residential areas (Ward 7 or Ward 8) for food shopping. Red circles represent the locations of grocery stores within the city boundaries of Washington, D.C. The three grocery stores located within the residential neighborhoods of Ward 7 and Ward 8 are demarcated as red dots within the Ward 7 and Ward 8 map boundaries. The red boundary line surrounds Washington, D.C., with the black lines demarcating the boundaries of the Washington, D.C., metropolitan area. Map created using QGIS, version 3.34.

As a result of the thematic analysis of the food shopping practices of the study participants, a map (Figure 1) was constructed to provide a display of the frequented food shopping locations within their residential areas (located within their respective residential ward) and the locations outside of their residential areas using a Q geographic information system (QGIS) version 3.34 and maps provided by Open Data DC [33].

### 2.7. Quantitative Data Analysis

Electronically administered survey topics included demographics, cooking behaviors, cooking self-efficacy, food agency, self-rated health, and food security status. Community-specific and cognitive interview validation of the cooking behavior survey utilized in Phase 1 has been described the study of [31]. In brief, the cooking behavior survey included questions on cooking skills, food skills, cooking frequency, time use for cooking, and social relationships connected to cooking [31]. Descriptive data from survey responses were analyzed and summary statistics (mean, standard deviation) were determined for continuous data responses. For categorical responses, percentages were determined from surveys. No statistical inference tests were conducted from survey response data. Scores were determined for the respective validated measures of cooking and food skills [34], food security [35], and food agency [36]. 

## 3. Results

### 3.1. Survey Results 

A total of 20 participants enrolled in five virtual focus groups that were conducted between March and April, 2021. All the recruited participants completed the online surveys and attended their scheduled focus groups. Focus groups were approximately 90 min each in duration. The mean age was 60.3 (SD 9.3) years old, with an age range of 34–72 years. All participants were U.S.-born, self-identified African American females, with 35% of the sample reporting full or part time employment (n = 7), while 50% reported being retired (n = 10). Half of the study population reported being married (n = 10) and 75% at least had a college degree (n = 15). Residents of Ward 7 comprised 80% (n = 16) of the study population. The most common CVD risk factor reported was that of being overweight or obese (n = 18). In terms of cooking behavior, the home cooking frequency of dinner was 4.0 times per week, which was representative of a moderate level of weekly cooking [3]. Both food and cooking skills, likewise, fell within the moderate skill levels as reported in the literature [34]. The majority of participants (n = 14) reported no food insecurity within the prior 12 months, and most participants had access to a personal vehicle for transportation (n = 14). Demographic characteristics of participants are provided in Table 1. 

### 3.2. Qualitative Results 

**Figure 2:**
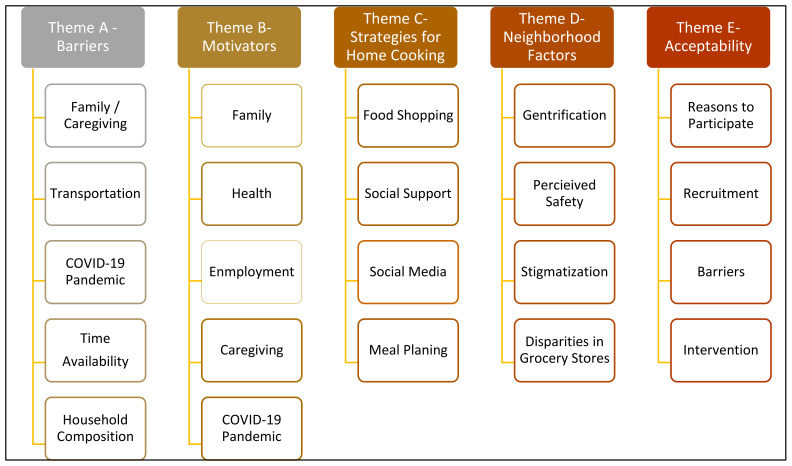
Overview of themes, subthemes and concepts.

Thematic analysis of focus group transcripts identified five main themes as follows: (A) barriers to cooking (family and caregiving, transportation, COVID-19 pandemic, time availability, household composition); (B) motivators to cooking (family, caregiving, health, enjoyment, COVID-19 pandemic); (C) strategies (food shopping, social support, social media, meal planning); (D) neighborhood (gentrification, perceived safety, stigmatization, disparities in grocery stores); (E) and acceptability of the intervention (reasons to participate, barriers, recruitment, intervention delivery) (Figure 2). Each theme and selected representative codes and quotes are described below and in Table 2. Codes and quotes were selected to be representative of the larger data set. All themes that are presented were discussed in all five focus groups. 

### 3.3. Barriers to Home Cooking 

Barriers to home cooking centered on the following six subthemes (codes): (1) family and caregiving, (2) transportation, (3) the COVID-19 pandemic, (4) time availability, and (5) household composition (Table 3). Lack of cooking skills or knowledge was not reported by focus group participants as a barrier to cooking. This may be indicated by the cooking skills and food skills scores among the participants. Motivators to home cooking included five subthemes (codes) as follows: (1) family, (2) health, (3) enjoyment, (4) caregiving, (5) the COVID-19 pandemic (Table 3). 

### 3.4. Motivators to Home Cooking

Motivators and barriers to cooking were not always distinctly different. There were some motivators that were reported by participants that could serve as barriers depending on time and planning factors. This included caregiving, the impact of the COVID pandemic, and the identification of time blocks during the day for cooking in the context of other activities. Instituting strategies and planning as well as personal experiences with cooking helped to differentiate when caregiving and time were either constructed as reasons to cook or as motivators. 

### 3.5. Strategies for Home Cooking

**Figure 3:**
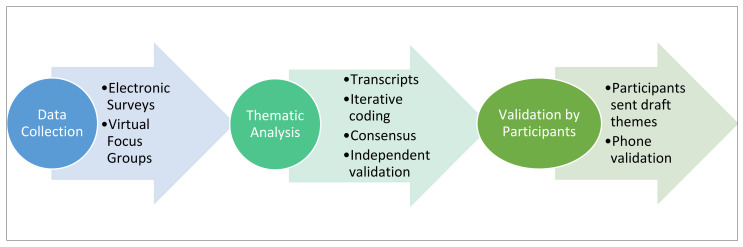
Data collection, thematic analysis, and qualitative data validation process.

The strategies for home cooking theme included the following subthemes (codes): (1) food shopping, (2) social support, (3) social media, and (4) meal planning (Table 2). Strategies related to participants’ residential neighborhoods centered on the provision of high quality and fresh foods outside of the residential neighborhood food environment. Participants reported traveling outside of their residential neighborhoods due to the lack of grocery stores at the time of the focus groups. Using the residential neighborhoods of Ward 7 and Ward 8 as reference points, a map of the locations used for food shopping as they were named by participants is provided in Figure 3. 

### 3.6. Neighborhood Factors

Participation in behaviors is influenced by an individual’s environment. Focus group participants commented on neighborhood conditions and factors that influence their food choices and dietary behaviors, including the following subthemes (codes): (1) gentrification, (2) perceived safety, (3) stigmatization, and (4) disparities in grocery stores (Table 2). Responses surrounding stigma were related to survey responses regarding the perception of the food environments within Ward 7 and Ward 8. 

### 3.7. Acceptability of the Intervention

With regard to the acceptability of the intervention, four subthemes (codes) emerged as follows: (1) reasons to participate, (2) study population, (3) barriers, and (4) intervention delivery (Table 2). The study design suggestions from the focus group participants included the targeted recruitment of young adult members of the community, focusing on organic foods and providing virtual class options. Suggested additions to the intervention classes included costs of recipe ingredients, using recipes with ingredient flexibility, nutrition information, and the provision or compensation of the ingredients used in virtual classes. The modifications of the intervention based on the focus group responses are presented in Table 3. 

### 3.8. Participant Check-in Validation Interview Results

Fourteen focus group participants (70%) consented to the validation interviews. All participants agreed on the presented themes and representative quotes. No additional themes or quotes were selected. Validation interviewees provided feedback on the temporal relevance of quotes and themes on food shopping and cooking behaviors from the time of the focus group. Relevant to neighborhood factors, participants told the research team during the validation interviews that one additional grocery store opened within Ward 8 from the time period of the focus groups to that of the validation interviews. Two participants stated that the grocery store location is within a previously developed commercial zone within the ward and may not be convenient for the individuals who lived in other sections of the ward. Other temporally related comments from the participants included increases in food prices, the conclusion of social distancing practices within grocery stores, and the return of individuals to an in-person work schedule as a result of vaccination and the decreasing severity of the COVID-19 pandemic. 

## 4. Discussion

Our sequential mixed methods study with self-identified African American women living within a low food access area within Washington, D.C., illustrated the role of individual, social, and food environmental contexts within perceptions of acceptability and participation in relation to the behavior of the research interest: cooking. The importance of community-tailored approaches for nutrition research among individuals from disparate populations has been widely reviewed. Tenets of community-tailored approaches overlay with a recently published framework that addresses the current gap in clinical nutrition interventions [11]. Namely, the framework calls for a focus on conducting exploratory studies, designing interventions that reflect current eating behaviors, and the consideration of the contextual variables that can mediate eating behaviors. The role of understanding current individual and family behaviors, as well as that of contextual variables, were paramount to our results. Participants in the virtual focus groups described barriers and motivators to cooking that were influenced by daily routines, household composition, and neighborhood environments. Perceptions of acceptability for the study were expressed based on the health needs of the community, the current food environment, and neighborhood factors such as gentrification. 

### 4.1. Comparison of Our Findings with the Established Literature 

Similar to previously published research, our study participants reported cooking dinner less than the majority of U.S. adults who report cooking dinner at least five times per week. Study participants reported cooking most often on the weekend and instituting time-saving strategies for meal planning such as batch cooking. Our study also found that barriers to cooking at home included household composition (number of people living in the household) and time availability. These findings are similar to those reported in prior studies of U.S. and non-U.S. adults [37,38,39,40,41]. Additionally, we identified motivators for cooking that were similar to those found in prior reports such as health considerations and enjoyment from cooking. Distinct from the prior literature on cooking interventions, our work identified a specific role for non-parental caregiving on cooking at home in terms of frequency and the types of foods cooked. Caregiving roles within our study population included caregiving for a spouse and extended family members including grandchildren. Although our overall study population was older and thus may not have had parental caregiving roles, the demands of caregiving in general with regard to a potential impact on cooking and meal planning have arisen in prior acceptability studies among parental populations [41,42]. Interestingly, caregiving was identified as both a motivator and barrier necessitating cooking, especially when a spouse had a health condition or if the participant wanted to introduce vegetables into the diet of grandchildren. Future cooking intervention studies may utilize these findings to specifically identify the role of caregiving or the types of caregiving roles on participation in cooking intervention studies. Additionally, interventions in the future may need to consider tailoring time requirements and intervention delivery based on non-parental caregiving roles within their study population. 

Our study focused on Ward 7 and Ward 8 of Washington, D.C., which are designated areas of low food access. Therefore, all the study participants would likely have limited access to non-processed or non-convenience-based foods. However, only 15% of the study participants reported experiencing food insecurity. This may be explained through the multi-dimensional nature of food insecurity. Food access is just one dimension of food insecurity. Other dimensions include availability and utilization [43,44]. Of note, the validated USDA food insecurity measure used within our study does not measure factors related to access and food environment. The measure focuses on outcomes, such as hunger, affordability, and weight changes that may occur when food access, availability, and utilization are low. Within our study sample, our qualitative results included strategies for home cooking and the provision of food that may impact utilization and availability despite limited geographic food access. 

In our results, the impact of the neighborhood environment on cooking behaviors focused not only on geographic food access but also on gentrification, perception of safety, customer service within residential stores, and issues with food, as well as produce quality and availability. The comparable impacts of the safety and quality of produce within low-access food neighborhoods food neighborhoods have been previously identified [15,45,46]. Gentrification, in particular, was brought up in all focus groups as a lynchpin issue impacting food availability and access. Gentrification is termed as the design of urban space for in-movers with the result of marginalizing the needs and rights of the existing residents [47]. Consistent with reports on how gentrification can influence food access, the focus group participants in our study reported the psychological impacts of gentrification, which included the newer markets established for newer residents and not for the use of current residents, thus still limiting food access. This psychological phenomenon has been reported in the literature and represents a nuanced understanding of access involving not only geographic but also social and economic dimensions [48]. 

In the wake of gentrification, the neighborhood environment reported by participants in all the focus groups was consistent with low-access to high-quality food retailers and increased access to convenience and fast-food retailers. This disparate geographic factor was discussed as the reason for the extension of their food shopping locations beyond their residential neighborhoods in order to meet their dietary needs. To our knowledge, prior formative research studies on cooking behaviors have not provided pre-intervention geospatial data on food shopping behavior as a component of an acceptability assessment. For our intervention purposes, understanding and detailing that pre-intervention grocery shopping was not limited to residential areas was important for several reasons. It removed ethical concerns that the intervention would force participants into logistically unfeasible new food provisioning routines. Furthermore, this understanding allows for the provision of food for the intervention sessions to be conducted from stores that participants currently utilize, thus likely optimizing the implementation of intervention recipes further. 

Acceptability is not solely an attribute of an intervention but is also a subjective evaluation made by individuals who expect to experience the intervention. As reported by Casale et al. [20], participant input from prospective acceptability-framed questions can include the intervention costs and barriers, perceived positive effects, relevance, acceptability to others, and potential self-efficacy. Similarly to Casale et al., we found that these factors were relevant. An optimal outcome from acceptability research is that of identifying the intervention modifications that will facilitate intervention feasibility and implementation, as well as those that will impact recruitment. Recruitment may be influenced when acceptability results outline particular dimensions related to frequency, curriculum components, timing of intervention sessions, purpose for participants, and specific members of the target population [19]. Our focus groups on acceptability allowed for the analysis of our planned intervention based on many of these dimensions. In particular, the need to recruit participants who were either younger in age or who were parents of young children were stated by the participants. This is not because cardiovascular disease risk factors would be present within this group, but the focus group members were, in fact, interested in the overall ability of a cooking intervention to prevent poor dietary quality and the attainment of risk factors among the young. Inter-generational concern regarding the impact of an adverse food environment on the dietary intake of young members of the community has been reported previously, including in relation to a study population living within a community analogous to Ward 7 and Ward 8 [49]. To address this concern, a response by the study team was to identify a location for the intervention sessions that would be accessible for parents of younger children and to provide an intervention schedule that could accommodate the parents of younger children by having weekend sessions with multiple time offerings. The type of intervention delivery was another important dimension of the acceptability discussed. The selection of a hybrid delivery method was recommended to both maintain social connections that are fostered through in-person settings and to provide an opportunity for implementing recipes within one’s own kitchen through virtual classes. The opinions provided by the focus group participants fit with recent observations in the literature on the effectiveness of virtual cooking interventions in dietary behaviors and health outcomes [50,51]. 

### 4.2. Implications for Future Cooking Intervention Community Studies 

Community-tailored research includes the concept of shared decision making to design and conduct the research. Modifying and adapting interventions based on participant’s behavior is an advantage with determining prospective acceptability. As highlighted in work previously conducted by Garcia et al. [9], Utter et al. [42], and Carman et al. [52], conducting formative research prior to an intervention initiation may positively impact intervention feasibility, retrospective acceptability, and attrition. An aspect of community-tailored research for qualitative studies includes the use of participant validation of results or participant check-in interviews. To our knowledge, our acceptability study is the first to document the use of participant check-ins within a community-engaged study on cooking behavior. 

### 4.3. Limitations 

There are several limitations to our study to consider. Despite recruitment being driven towards all self-identified genders, only female participants responded and enrolled to participate in the focus groups. This fundamental limitation in the external validity of our study findings may stem from the use of the umbrella CBPR study for recruitment as this study is predominately composed of self-identified females. As a result of only having one gender represented, our data do not include the diversity of social contexts with respect to gender, including a male view of caregiving and dietary health concerns. In comparison with the general population of Ward 7 and Ward 8, our study population differed with regard to age and economic level. Our study population was older, with a mean age of 60.3 years, which differs from the median age within both Ward 7 and Ward 8. The majority of our study population also reported having personal transportation access, which also differs from the majority of the population within both wards [53]. Lastly, with regard to demographic comparisons, 35% of the study population reported an annual household income level of at least $70,000, which means that approximately a third of the study participants were markedly above the median household income for both wards. Our study results may also have limited applicability to populations who do not report confidence with cooking and food skills. The majority of our participants reported being skilled in food skills and cooking skills at home. Therefore, our acceptability determination and possibly the impact on the utilization of food with regard to food security may be based on these lived experiences. Our virtual focus groups also did not include a sampling of foods from the curriculum recipes. Thus, discussions stemming from the experience of tasting specific regional or ethnic-based cultural flavors were potentially limited. Despite these limitations, our analysis does contain significant strengths. As discussed in Ayala et al. [54], focus groups have an advantage of providing participants with an environment for collective brainstorming on the research topic, thus creating a “synergistic group effect”. Furthermore, our study employed the use of participant interviews to validate thematic analysis findings. This methodology has been found to promote and instill trustworthiness between research teams and community members. 

## 5. Conclusions

Our study assessed the prospective acceptability of a community-tailored cooking intervention of D.C. COOKS. Our results showed that the planned intervention should be modified to address the health needs of the community and, in particular, to deliver intervention that is modified to leverage the benefits from both in-person delivery and virtual delivery. Additionally, an understanding of pre-intervention behaviors allowed for a contextual understanding of how dietary behaviors from the intervention may be sustainably implemented. 

## Figures and Tables

**Table 1:** Phase 1 focus group participant demographic, cardiovascular risk factor, and food security characteristics, n = 20.

	Mean (SD)
Age	60.3 (9.3)
Sex (%)	n (%)
Female	20 (100)
Annual household income	n (%)
≤$29,999	7 (35)
$30,000–$69,999	6 (30)
≥$70,000	7 (35)
Household size	1.8 (1.4)
Weekly frequency of cooking dinner	4.0 (2.4)
Food skill score	47.4 (10.8)
Cooking skill score	29.8 (6.1)
Cardiovascular risk factor (* not mutually exclusive)	n (%)
Obesity/overweight	18 (90)
Hyperlipidemia	9 (45)
Hypertension/pre-Hypertension	14 (70)
Type 2 diabetes/pre-diabetes	4 (20)
Current or prior smoker (any type)	8 (40)
Employment status	n (%)
Full-time	5 (25)
Part-time	2 (10)
Unemployed	3 (15)
Retired	10 (50)
Marital status	n (%)
Married	10 (50)
Single	4 (20)
Divorced/widowed	6 (30)
Education status	n (%)
Some college education and below	5 (25)
College degree	8 (40)
Graduate or post-graduate degree	7 (35)
Food security status	n (%)
Full food security	14 (70)
Marginal food security	3 (15)
Low food security	1 (5)
Very low food security	2 (10)
Personal transportation access	n (%)
Drive	14 (70)
Not drive	6 (30)
Age, started to cook	13 (3.9)
Use of batch cooking at home **	n (%)
Yes	12 (60)
No	8 (40)

* Maximum food skills score of 65 and maximum cooking skills score of 45. ** Batch cooking is defined as cooking particular amounts of food at one time that are intended for later consumption.

**Table 2:** Themes and subthemes with selected codes and quotes.

Theme A: Barriers to Home Cooking				
**A1: Family and Caregiving** “I was saying, for me, there are some challenges. The first is I have young kids. And so, it’s a time and energy. Sometimes I’m just like tired. And in particular since the pandemic and being home with the kids all the time, I’m feeling like I am always in the kitchen.”	**A2: Transportation** “By the time you go to the store and get back, I feel like—you might not feel like cooking what you planned on cooking”…“That might happen, maybe, I would say, within a week, I am going. maybe once out of a week. Seven days, once out of the week. I catch the bus, two bus—more so two buses, yes. Maybe we’ll go down the street to safeway and bring it on the bus. And it’s what I planned to eat that day, you know, and I just don’t feel like it.” “With me, before and during the pandemic, uhm getting to the stores because I don’t drive. So, therefore, you know, even walking, catching the bus, and get into particular stores that I want to go to where I will have that ask someone to take me.”	**A3: COVID-19 Pandemic** “Like why is somebody always asking me (to cook)—like the worst question you can ask me, especially if I didn’t have a plan is what’s for lunch or what’s for dinner. Didn’t I just feed you? They’re like no. I just fed you. I was just in the kitchen, yeah. So, that has been extremely challenging, very frustrating.”	**A4: Time Availability** “By the time I’m coming to cook, it’s like—and even when I go to Costco, I usually bring a pizza home, I’m not even going to lie. Because you’ve done all that work. We all know. [unintelligible] the job, the planning, the getting the stuff, standing in line. Then, by the time you get home, it’s like, I’ve got to put these together to make a meal? [laugh]” “But with my timing and if I haven’t cooked by, have dinner ready by 5:00 you can forget it. You better be on your way out to get something.”	**A5: Household Composition** “Because eating by yourself, I don’t have any problems with it, but I think I would be more apt to cook if, it was more than just me. You know, because I waste a lot of food, too. Because a lot of times, I can’t really prepare for one. Sometimes, depending on what you’re cooking, for example, spaghetti. Not that I make a huge pot of what I make. (But) after two times, I’m done. I really don’t think about freezing and that kind of thing.”
**Theme B: Motivators to Home Cooking**				
**B1: Family and Sharing** “As much as I do enjoy cooking, it’s not about the cooking per se. The enjoyment comes from cooking food that other people enjoy and appreciate. So, I don’t know if I love cooking as much as I do love cooking for people who are like foodies, who are going to appreciate like, “Oh man, I can taste this,” or like people who can get into the experience of the food with me is more enjoyable than just cooking. Like the art of it to me is both the cooking and the partaking of the meal.”	**B2: Health** “Because when you’re cooking at home, you know what’s coming, it’s not just flavor, but if you have high blood pressure, you’re always monitoring your sodium intake. So, uhm, cooking at home, at least you know. At least you know what you’re putting in and stuff and so, yeah.”	**B3: Enjoyment** “For everything, you use your mind and your hands, and you know, it’s not about the measuring spoons when you cook, you know, you just have your own way of doing it and it still comes out right.” “You could create your own recipes, or you can modify like certain things I don’t eat, I may not put it in that particular dish, or, you know…, take something out, put something in, season it to your taste. Because most recipes have all the spices you’re supposed to use, I’ll add something a little different—lemon pepper versus regular pepper, something like that.”	**B4: Caregiving** “As much as I do enjoy cooking, it’s not about the cooking per se. The enjoyment comes from cooking food that other people enjoy and appreciate. So, I don’t know if I love cooking as much as I do love cooking for people who are like foodies, who are going to appreciate like, “Oh man, I can taste this,” or like people who can get into the experience of the food with me is more enjoyable than just cooking. Like the art of it to me is both the cooking and the partaking of the meal.”	**B5: COVID-19 Pandemic** “Since this pandemic, I kind of changed my outlook on cooking. I do more of it now than what I did in the past. I will cook probably three to four times a week, and I do more planning because that’s my biggest issue is planning. If I plan, I can do it.”
**Theme C: Strategies for Home Cooking**				
**C1: Food Shopping** “We’re living in Ward 7 with only one supermarket. And I have to go out of my neighborhood to go grocery shopping. Not that I don’t go to that place very occasionally but I hate to have to go out of my neighborhood but I do.”	**C2: Social Support** “So my friends, one of my ministers, we talk about sharing recipes. So we try each other’s recipes. And so that kind of motivates you to do something a little bit different.”	**C3: Social Media** “Well, another site for great recipes, Pinterest, they have a whole lot of good stuff there. One of my great favorite is the noodles and then I had a dish of um, like, a seven-bean um, salad. Once you cook your noodles and rinse them, and then add the seven-bean salad to it, and then heat it up at very low temperature and add your favorite sauce of any kind, it might be, for example, a chili, um green chili sauce. Oh, my husband just laid that out. ‘Oh, this was really good.’”	**C4: Meal Planning** “I just like to cook or I plan, even when I’m laying down thinking the next day of everything I need to do and how to ah… juggle it all, and… now with someone coming, they’re going wanna eat, so I cook.”	
**Theme D: Neighborhood Factors**				
**D1: Gentrification** “And the people that do care, there like they said, they just want to get our property and get (us) away. Like I say, it’s ridiculous how D.C. treats the people who were here when it was—the older people and the original people who were here, who stayed here through all of the mess and dealt with the foolishness and how they don’t look out for the people who are here.”	**D2: Perceived Safety** “I’m not going to lie, I’m nervous to get out the car. When I’m going to the neighborhood corner store, that would be for soda or something unhealthy. I’m looking for real groceries to feed my family.”	**D3: Stigmatization** “In this city, that’s what they think of Ward 7 and 8 and stigmatize it a lot.” (Referring to questions on a survey that were skipped by some participants). “Hypothetically, thinking, perhaps the reason why they were skipped is because that people have a lot of hidden agendas when you go into these neighborhoods. And like I said gentrification is here, and it’s real. And perhaps they don’t know who going to get hold to this information, this survey, and the reason why… But we don’t know where this information is going to go to. And people always have hidden agendas when you come into a community that’s full of Black and Brown people”	**D4: Disparities in Grocery Stores** “Yeah. It’s very true. Or even going, like she said, across the bridge. I work on 14th St. I shop at the (X Grocery store name) right there. It’s a total difference than when you go to Alabama Ave., which has its own issues. You don’t know if you’re going to honestly go in there and get robbed or shot or you’re going to come out with quality food or is it going to last.”	
**Theme E: Acceptability of the Intervention**				
**E1: Reasons to Participate** “I can think of quite a few people that would be interested in that. And, and it would be a good thing for those who want to change their health condition or health situation. So they can enjoy their food even more. And some people cook and they say they don’t like how their food tastes but they’d rather have someone else’s food.” “Ward 7 needs it. I’m concerned about my people. They need to stay out of McDonalds.” “I think would they enjoy… whoever is getting the class—they would enjoy learning how to cook different things.”	**E2: Recruitment** “I would also think about involving more people that are like young adults. We have children now. The other two ladies and myself, you know, we’re retired or, you know, older. So, our cooking may be different. But younger adults, young families, making sure that they have a better understanding of preparing nutritional meals for their children because I’m seeing—I have a friend that works in the school system and sometimes I go there to volunteer. And the children seem to not have an appreciation of the lunch, you know. They pick over things and then throw them away. You know, so, maybe more young adults with families.”	**E3: Barriers** “The costs typically associated with healthier food options. And that definitely can be a hindrance, it never make sense to me that the healthier food options cost more than the unhealthy stuff when, you know, it should be the flip side. And so, I guess I’d love as a part of this conversation or part of your study if we spent some time to get some like tricks to getting cost saving healthy items including produce organic, when necessary.”	**E4: Intervention** (Regarding the study)” Is it going to be face to face, or is it going to be virtual, and (what is) the time period that you are going to use?” (Referencing prior virtual cooking class) “And she (chef) out all the things that we need, she sent out the recipe that we were going to be cooking and whatever and she demonstrated and whatever, it was really good too, so. Because we was on Zoom and in our kitchen… And it was really good because you saw other people cooking and giving their feedback and whatever.” “Yes. I guess I assumed that either ingredients would be provided or gift card to get the items like a list of shopping items that we will have to get.” “I only have a kitchenette so—space, you know. That’s why I say, how simple would it be? Will I be all in the dining room, in the living room trying to be all over the place cooking because my kitchen is so small?”	

**Table 3:** Modifications to the cooking intervention of D.C. COOKS based on focus group participant suggestions.

Component of Phase 2 D.C. COOKS	Summarized Suggestions from the Focus Groups	Planned Modifications	Related Participant Barrier, Motivator, or Strategy
**Recipes**	Provide nutritional information for recipes, including sodium content. Provide information on potentially unhealthful ingredients that people may use and showcase where more healthful options are used. Provide cost information.	Interventionist will provide information during classes highlighting healthful substitutions made within recipes compared with unhealthy ones. Nutritional information for recipes were planned but will be highlighted as part of the shared meal discussion during classes, including the use of organic foods. Cost information for ingredients will be provided in class.	Health concerns of participant and family members. Economic concerns directly from food pricing or from indirect costs related to traveling for food shopping.
**Delivery of intervention**	Provide in-person and virtual options for participants.	Hybrid design for classes will occur with three in-person and thre virtual classes.	Reduced barrier for time and transportation concerns to community site to promote the interest of family and household members.
**Community site or setting**	Choose location known to study participants and that is easily accessible.	Community kitchen site was discussed with participants during validation interviews and the majority of participants were knowledgeable of the site and agreed on its accessibility for those who have personal transportation. Transportation for those with personal needs will be provided to and from the community site.	Community support network as a motivator for cooking, with a reduction in transportation and safety. concerns.
**Food supplies to participants**	Provide ingredients for recipes and assist with the measured amounts needed.	Research team will utilize food pantry infrastructure within the community kitchen site to provide ingredients for virtual sessions. Recipes for these sessions were selected based on seasonal availability through the community site. The community site will be the pick-up location for ingredients for the three weeks of virtual classes.	Access and availability of foods and reducing the need to travel for ingredients.
**Cooking behavior diaries**	Understand the importance of motivators and barriers to cooking at home.	Questions asked through a diary regarding enjoyment, use of creativity, and people whom participants cooked with or for were added.	Inquire about participants’ stated motivators and barriers for cooking at home.
**Grocery store receipts**	Privacy and feasibility concerns regarding submitting food receipts to the research team due to many participants using electronic receipt phone applications.	Research questions will ask for a self-report of the prior 30-day food expenditures at grocery stores.	Avoid privacy and logistic issues reported by participants that would increase participant burden.

## Data Availability

The datasets presented in this article are not readily available due to inclusion in an ongoing clinical protocol. The raw data supporting the conclusions of this article will be made available by the authors on request.

## Supplementary Materials

The following supporting information can be downloaded at: https://www.mdpi.com/article/10.3390/nu16030463/s1, Focus Group Moderator Guide.

## References

[1] GBD 2017 Risk Factor Collaborators (2018). Global, regional, and national comparative risk assessment of 84 behavioural, environmental and occupational, and metabolic risks or clusters of risks for 195 countries and territories, 1990–2017: A systematic analysis for the Global Burden of Disease Study 2017. Lancet.

[2] Li Y., Xia P.-F., Geng T.-T., Tu Z.-Z., Zhang Y.-B., Yu H.-C., Zhang J.-J., Guo K., Yang K., Liu G. (2023). Trends in Self-Reported Adherence to Healthy Lifestyle Behaviors Among US Adults, 1999 to March 2020. JAMA Netw. Open.

[3] Wolfson J.A., Leung C.W., Richardson C.R. (2020). More frequent cooking at home is associated with higher Healthy Eating Index-2015 score. Public Health Nutr..

[4] Farmer N., Wallen G.R., Yang L., Middleton K.R., Kazmi N., Powell-Wiley T.M. (2019). Household Cooking Frequency of Dinner Among Non-Hispanic Black Adults is Associated with Income and Employment, Perceived Diet Quality and Varied Objective Diet Quality, HEI (Healthy Eating Index): NHANES Analysis 2007–2010. Nutrients.

[5] Reicks M., Gold A., Tran N., LeBlanc K. (2022). Impacts of A Taste of African Heritage: A Culinary Heritage Cooking Course. J. Nutr. Educ. Behav..

[6] Greenlee H., Gaffney A.O., Aycinena A.C., Koch P., Contento I., Karmally W., Richardson J.M., Lim E., Tsai W.Y., Crew K. (2015). ¡Cocinar Para Su Salud!: Randomized Controlled Trial of a Culturally Based Dietary Intervention among Hispanic Breast Cancer Survivors. J. Acad. Nutr. Diet..

[7] Condrasky M.D., Baruth M., Wilcox S., Carter C., Jordan J.F. (2013). Cooks training for Faith, Activity, and Nutrition project with AME churches in SC. Eval. Program Plan..

[8] Schoenberg N.E., Howell B.M., Swanson M., Grosh C., Bardach S. (2013). Perspectives on healthy eating among Appalachian residents. J. Rural. Health.

[9] Garcia T., Ford B., Pike D., Bryce R., Richardson C., Wolfson J.A. (2021). Development and implementation of a community health centre-based cooking skills intervention in Detroit, MI. Public Health Nutr..

[10] Lichtenstein A.H., Appel L.J., Vadiveloo M., Hu F.B., Kris-Etherton P.M., Rebholz C.M., Sacks F.M., Thorndike A.N., Van Horn L., Wylie-Rosett J. (2021). 2021 Dietary Guidance to Improve Cardiovascular Health: A Scientific Statement From the American Heart Association. Circulation.

[11] Dhillon J., Jacobs A.G., Ortiz S., Diaz Rios L.K. (2022). A Systematic Review of Literature on the Representation of Racial and Ethnic Minority Groups in Clinical Nutrition Interventions. Adv. Nutr..

[12] Tewahade S., Berrigan D., Slotman B., Stinchcomb D.G., Sayer R.D., Catenacci V.A., Ostendorf D.M. (2022). Impact of the built, social, and food environment on long-term weight loss within a behavioral weight loss intervention. Obes. Sci. Pract..

[13] Powell-Wiley T.M. (2023). Centering Patient Voices Through Community Engagement in Cardiovascular Research. Circulation.

[14] Powell-Wiley T.M., Baumer Y., Baah F.O., Baez A.S., Farmer N., Mahlobo C.T., Pita M.A., Potharaju K.A., Tamura K., Wallen G.R. (2022). Social Determinants of Cardiovascular Disease. Circ. Res..

[15] Zenk S.N., Lachance L.L., Schulz A.J., Mentz G., Kannan S., Ridella W. (2009). Neighborhood retail food environment and fruit and vegetable intake in a multiethnic urban population. Am. J. Health Promot..

[16] Castelo A.F.M., Schäfer M., Silva M.E. (2021). Food practices as part of daily routines: A conceptual framework for analysing networks of practices. Appetite.

[17] Raber M., Wolfson J. (2021). The Challenging Task of Measuring Home Cooking Behavior. J. Nutr. Educ. Behav..

[18] Mills S., White M., Brown H., Wrieden W., Kwasnicka D., Halligan J., Robalino S., Adams J. (2017). Health and social determinants and outcomes of home cooking: A systematic review of observational studies. Appetite.

[19] Gooding K., Phiri M., Peterson I., Parker M., Desmond N. (2018). Six dimensions of research trial acceptability: How much, what, when, in what circumstances, to whom and why?. Soc. Sci. Med..

[20] Casale M., Somefun O., Haupt Ronnie G., Desmond C., Sherr L., Cluver L. (2023). A conceptual framework and exploratory model for health and social intervention acceptability among African adolescents and youth. Soc. Sci. Med..

[21] Sekhon M., Cartwright M., Francis J.J. (2017). Acceptability of healthcare interventions: An overview of reviews and development of a theoretical framework. BMC Health Serv. Res..

[22] Reimers T.M., Wacker D.P. (1988). Parents’ Ratings of the Acceptability of Behavioral Treatment Recommendations Made in an Outpatient Clinic: A Preliminary Analysis of the Influence of Treatment Effectiveness. Behav. Disord..

[23] Yingling L.R., Brooks A.T., Wallen G.R., Peters-Lawrence M., McClurkin M., Cooper-McCann R., Wiley K.L., Mitchell V., Saygbe J.N., Johnson T.D. (2016). Community engagement to optimize the use of web-based and wearable technology in a cardiovascular health and needs assessment study: A mixed methods approach. JMIR mHealth uHealth.

[24] Thomas S., Yingling L., Adu-Brimpong J., Mitchell V., Ayers C.R., Wallen G.R., Powell-Wiley T.M. (2017). Mobile health technology can objectively capture physical activity (PA) targets among African-American women within resource-limited communities—The Washington, DC cardiovascular health and needs assessment. J. Racial Ethn. Health Disparities.

[25] Farmer N., Powell-Wiley T.M., Middleton K.R., Roberson B., Flynn S., Brooks A.T., Kazmi N., Mitchell V., Collins B., Hingst R. (2020). A community feasibility study of a cooking behavior intervention in African-American adults at risk for cardiovascular disease: DC COOKS (DC Community Organizing for Optimal culinary Knowledge Study) with heart. Pilot Feasibility Stud..

[26] Farmer N., Tuason R.T., Kazmi N., Flynn S., Mitchell V., Middleton K., Cox R., Franklin K., Gordon T., Baginski A. (2022). Going virtual during the COVID-19 pandemic: Adaptation of a mixed-methods dietary behavior study within a community-based participatory research study of African-American adults at risk for cardiovascular disease. BMC Med. Res. Methodol..

[27] D.C. Health Matters, Ward 7. https://www.dchealthmatters.org/demographicdata?id=131494.

[28] D.C. Health Mattes, Ward 8. https://www.dchealthmatters.org/demographicdata?id=131495.

[29] King C.J., Buckley B.O., Maheshwari R., Griffith D.M. (2022). Race, Place, And Structural Racism: A Review Of Health And History in Washington, D.C. Health Aff..

[30] (2014). DC Health Department Report. Obesity in the District of Columbia. https://dchealth.dc.gov/publication/obesity-report-2014.

[31] Farmer N., Powell-Wiley T.M., Middleton K.R., Brooks A.T., Mitchell V., Troncoso M., Ceasar J., Claudel S.E., Andrews M.R., Kazmi N. (2022). Use of a focus group-based cognitive interview methodology to validate a cooking behavior survey among African-American adults. Front. Nutr..

[32] Ames N.J., Peng C., Powers J.H., Leidy N.K., Miller-Davis C., Rosenberg A., VanRaden M., Wallen G.R. (2013). Beyond intuition: Patient fever symptom experience. J. Pain Symptom Manag..

[33] Open Data DC. https://opendata.dc.gov/.

[34] Lavelle F., McGowan L., Hollywood L., Surgenor D., McCloat A., Mooney E., Caraher M., Raats M., Dean M. (2017). The development and validation of measures to assess cooking skills and food skills. Int. J. Behav. Nutr. Phys. Act..

[35] United States Department of Agriculture Household Food Security Module. https://www.ers.usda.gov/topics/food-nutrition-assistance/food-security-in-the-u-s/survey-tools/#household.

[36] Lahne J., Wolfson J.A., Trubek A. (2017). Development of the Cooking and Food Provisioning Action Scale (CAFPAS): A new measurement tool for individual cooking practice. Food Qual. Prefer..

[37] Wolfson J.A., Ramsing R., Richardson C.R., Palmer A. (2019). Barriers to healthy food access: Associations with household income and cooking behavior. Prev. Med. Rep..

[38] Bisogni C.A., Jastran M., Shen L., Devine C.M. (2005). A biographical study of food choice capacity: Standards, circumstances, and food management skills. J. Nutr. Educ. Behav..

[39] Blake C.E., Bisogni C.A., Sobal J., Jastran M., Devine C.M. (2008). How adults construct evening meals. Scripts for food choice. Appetite.

[40] Liu B., Widener M.J., Smith L.G., Farber S., Gesink D., Minaker L.M., Patterson Z., Larsen K., Gilliland J. (2022). Who’s cooking tonight? A time-use study of coupled adults in Toronto, Canada. Time Soc..

[41] Laila A., Leme A.C., Hou S., Ma D.W.L., Haines J. (2023). Perceived challenges and strategies to achieve Canada’s Food Guide recommendation to “Cook more often”: Findings from parents of young children. Appetite.

[42] Utter J., Larson N., Berge J.M., Eisenberg M.E., Fulkerson J.A., Neumark-Sztainer D. (2018). Family meals among parents: Associations with nutritional, social and emotional wellbeing. Prev Med..

[43] Ashby S., Kleve S., McKechnie R., Palermo C. (2016). Measurement of the dimensions of food insecurity in developed countries: A systematic literature review. Public Health Nutr..

[44] Bartelmeß T., Jasiok S., Kühnel E., Yildiz J. (2022). A scoping review of the social dimensions in food insecurity and poverty assessments. Front. Public Health.

[45] Bader M.D.M., Purciel M., Yousefzadeh P., Neckerman K.M. (2010). Disparities in Neighborhood Food Environments: Implications of Measurement Strategies. Econ. Geogr..

[46] Gie S., Borthwick F. (2013). Gentrification and Food Environments: A Rapid Evidence Assessment. medRxiv.

[47] Ong V., Skinner K., Minaker L.M. (2021). Life stories of food agency, health, and resilience in a rapidly gentrifying urban centre: Building a multidimensional concept of food access. Soc. Sci. Med..

[48] Tran L.D., Rice T.H., Ong P.M., Banerjee S., Liou J., Ponce N.A. (2020). Impact of gentrification on adult mental health. Health Serv. Res..

[49] Hines A.L., Brody R., Zhou Z., Collins S.V., Omenyi C., Miller E.R., Cooper L.A., Crews D.C. (2022). Contributions of Structural Racism to the Food Environment: A Photovoice Study of Black Residents With Hypertension in Baltimore, MD. Circ Cardiovasc. Qual Outcomes.

[50] Lillquist S., Ruiz Barnecett G., Flexman N., Mikati N. (2022). Recipes for Health: A Community-Based Nutrition and Culinary Intervention. Cureus.

[51] Sharma S.V., McWhorter J.W., Chow J., Danho M.P., Weston S.R., Chavez F., Moore L.S., Almohamad M., Gonzalez J., Liew E. (2021). Impact of a Virtual Culinary Medicine Curriculum on Biometric Outcomes, Dietary Habits, and Related Psychosocial Factors among Patients with Diabetes Participating in a Food Prescription Program. Nutrients.

[52] Carman K., Sweeney L.H., House L.A., Mathews A.E., Shelnutt K.P. (2021). Acceptability and Willingness to Pay for a Meal Kit Program for African American Families with Low Income: A Pilot Study. Nutrients.

[53] D.C. Policy Center (2019). Food Access in D.C is Deeply Connected to Poverty and Transportation.

[54] Ayala G.X., Elder J.P. (2011). Qualitative methods to ensure acceptability of behavioral and social interventions to the target population. J. Public Health Dent..

